# Chlorophyll fluorescence imaging captures photochemical efficiency of grain sorghum (*Sorghum bicolor*) in a field setting

**DOI:** 10.1186/s13007-020-00650-0

**Published:** 2020-08-10

**Authors:** Matthew T. Herritt, Duke Pauli, Todd C. Mockler, Alison L. Thompson

**Affiliations:** 1 U.S. Department of Agriculture, Agricultural Research Service, U.S. Arid-Land Agricultural Research Center, Maricopa, AZ 85138 USA; 2 The School of Plant Sciences, University of Arizona, Tucson, AZ 85721 USA; 3 Donald Danforth Plant Science Center, Saint Louis, MO 63132 USA

**Keywords:** Chlorophyll fluorescence, Sorghum, Chlorophyll fluorescence imaging

## Abstract

**Background:**

Photosynthesis is one of the most important biological reactions and forms the basis of crop productivity and yield on which a growing global population relies. However, to develop improved plant cultivars that are capable of increased productivity, methods that can accurately and quickly quantify photosynthetic efficiency in large numbers of genotypes under field conditions are needed. Chlorophyll fluorescence imaging is a rapid, non-destructive measurement that can provide insight into the efficiency of the light-dependent reactions of photosynthesis.

**Results:**

To test and validate a field-deployed fluorescence imaging system on the TERRA-REF field scanalyzer, leaves of potted sorghum plants were treated with a photosystem II inhibitor, DCMU, to reduce photochemical efficiency (F_V_/F_M_). The ability of the fluorescence imaging system to detect changes in fluorescence was determined by comparing the image-derived values with a handheld fluorometer. This study demonstrated that the imaging system was able to accurately measure photochemical efficiency (F_V_/F_M_) and was highly correlated (*r* = 0.92) with the handheld fluorometer values. Additionally, the fluorescence imaging system was able to track the decrease in photochemical efficiency due to treatment of DCMU over a 7 day period.

**Conclusions:**

The system’s ability to capture the temporal dynamics of the plants’ response to this induced stress, which has comparable dynamics to abiotic and biotic stressors found in field environments, indicates the system is operating correctly. With the validation of the fluorescence imaging system, physiological and genetic studies can be undertaken that leverage the fluorescence imaging capabilities and throughput of the field scanalyzer.

## Background

Sorghum [*Sorghum bicolor* (L.) Moench] is currently the fifth most important cereal crop in the world and is used for fuel, feed, and food. Global exports of sorghum rose from 3.56 to 3.68 million tons from 2018 to 2019, with the United States providing ~ 65% of exports in both years (2.30 to 2.50 million tons, respectively) (USDA Grain: World Markets and Trade https://www.fas.usda.gov/data/grain-world-markets-and-traded). Because sorghum originated in North Africa, it favors high temperatures (> 25 °C), and can tolerate prolonged periods of drought, making it an ideal crop for resource-limited production environments [1]. As the global population continues to rise, sorghum production must steadily increase to meet the demands for a low-cost, high nutrient cereal for non-industrialized countries [2]. However, as with all crops, changing weather patterns and increased water scarcity threaten global sorghum production. To address this challenge, new technologies are needed to better understand the physiology of key traits underlying sorghum’s growth and development so that they can be harnessed for both use in basic research as well as application to cultivar development.

Photosynthesis is an important physiological process that enables plants to convert solar radiation into chemical energy in the form of biomass [3–6]. Light is absorbed by antenna pigments (chlorophyll) and the excitation energy is transferred to photosystems. The energy transfer drives photochemical reactions (photosynthesis) that enable biomass accumulation. While this process is very important for plant growth, it is also highly inefficient. It is estimated that C_4_ plants, grown under optimal field conditions, only utilize 3% of the incoming solar radiation for photosynthesis while C_3_ plants use less than 3% [7–10]. After light energy is trapped in the reaction centers, but before respiratory processes, the minimal energy loss of C3 plants due to electron transport and carbohydrate assimilation has been calculated to be 24.6% of the total incoming solar radiation [3]. Thus, improving the efficiency of electron transport and carbohydrate assimilation could increase the radiation use for C3 plants. The remaining light absorbed by chlorophyll can either be dissipated as heat or released as fluorescence. As photosynthesis, heat dissipation, or fluorescence are the only three possible outcomes for chlorophyll absorbed light energy, measuring one can provide information about the other two.

Advances in measuring leaf-level fluorescence have made capturing chlorophyll fluorescence an important tool for studies focused on radiation-use efficiency and changes in photosynthesis. Measurements of chlorophyll fluorescence are rapid, non-invasive, and have allowed researchers to obtain information about how photosynthesis responds to heat stress [11, 12], water stress [13, 14], nitrogen deficiency [15, 16] and high light [17]. Herbicides like 3-(3′,4′-dichlorophenyl)-1,1-dimethylurea (DCMU), which interact with the D1 protein of photosystem II and block electron transport, has allowed researchers to better understand how chlorophyll fluorescence relates to the light-dependent reactions of photosynthesis [18–21]. Maximum fluorescence (F_M_) is associated with the reduction of plastoquinone. This was determined by showing that DCMU blocks electron transfer from photosystem II to plastoquinone which resulted in depressed maximum fluorescence (F_M_) signals [22]. The interaction between DCMU and the D1 protein of photosystem II directly impacts the light-dependent reactions of photosynthesis [23]. The effects DCMU has on photosystem II and chlorophyll fluorescence have been well reported [24, 25]; therefore DCMU has been used in numerous studies to validate the ability of fluorescence imaging systems to detect changes in fluorescence levels related to photosynthetic efficiency [26–28].

While many advances have improved understanding of how photosynthesis and chlorophyll fluorescence respond to environmental stressors, these measurements are still not readily adopted into large breeding trials for crop improvement. This is largely due, in part, to how traditional measurements of chlorophyll fluorescence are captured. The throughput of handheld fluorometer measurement systems (i.e., number of measurements per unit of time) is limited by how quickly the system can be transported from one plant to the other [29]. Also, the measurements from these instruments only integrate the fluorescence from a small leaf area which excludes most of the plot level information. These logistical and technical constraints make collecting chlorophyll fluorescence from large populations of field-grown plants difficult and only provides information on a very small area of the plot’s canopy.

Chlorophyll fluorescence imaging systems allow for rapid, non-contact measurements of photosynthesis. Early research utilizing fluorescence imaging systems were developed to investigate the spatial response of photosynthesis over a leaf to different diseases [30–32]. Utilization of fluorescence imaging systems allows for full canopy fluorescence information to be obtained from photosynthetic tissue. Thus, chlorophyll fluorescence imaging systems have been used for screening large populations to identify mutant photosynthetic phenotypes [33, 34], disease tolerance [35, 36] and freeze damage [37]. The design of fluorescence imaging systems has many common components.

Chlorophyll fluorescence imaging systems are composed of several essential components: camera, light source, filters and control system. Early imaging systems leveraged the characteristic that fluorescence is re-emitted as longer wavelengths, 650–800 nm [38]. Thus, filters are used to allow only light within the range of chlorophyll fluorescence (650–800 nm) to enter the camera and be used for quantitation. To measure F_M_ the plant tissue needs to be exposed to a saturating amount of light (> 3000 µmol) in a very short time (~ 1 s). Depending on the camera’s field of view (FOV), a light source that can provide the required amount of light homogenously within the camera’s FOV must be used. More recently, companies such as Qubit Phenomics (Ontario, Canada), Photon Systems Instruments (Drasov, Czech Republic), and PhenoVation (Wageningen, Netherlands) have provided chlorophyll fluorescence imaging systems that incorporate panels of light-emitting diodes (LEDs) which can provide this saturating pulse of light to the area within the FOV of the camera being used. Chlorophyll fluorescence imaging systems are generally deployed in laboratory or controlled environments, where camera settings and plant placement are optimized, producing high-quality data.

To improve plant photosynthesis, fluorescence imaging data need to be obtained from field-grown plants. The goal of the present study was to validate a PS2 fluorescence imaging system deployed on a large, outdoor, gantry-based phenotyping system (the TERRA REF field scanalyzer) with a commercial handheld fluorometer. To achieve this overall goal, the objectives of the present study were to: (1) compare the fluorescence phenotypes obtained from a handheld fluorometer with the gantry-based imaging system; (2) determine if the imaging system can capture the dynamic response of leaves treated with DCMU, a known inhibitor of photosystem II, over time; and (3) discuss the effectiveness of this system for phenotyping large genetic populations compared to the handheld fluorometer.

## Results

### Correlation between handheld and imaging

**Fig. 1:**
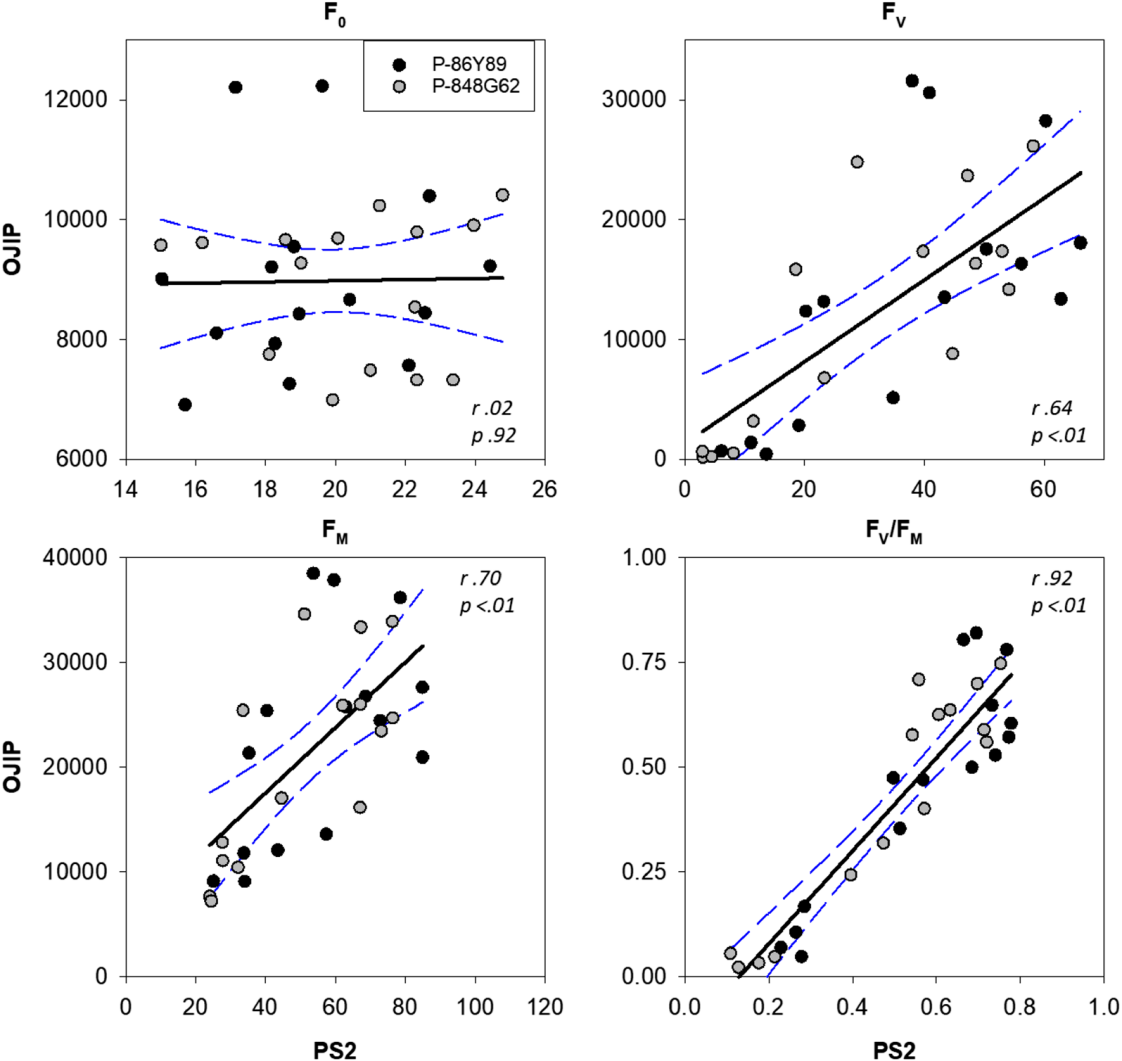
Scatter plots of handheld fluorescence (OJIP) versus imaging fluorescence (PS2). Circles represent the average values for each Day X Treatment (N = 4). The black lines represent regression with associated Pearson’s correlation (*r*), and *p* value (*p*) calculated from the SAS CORR procedure in the upper right corner. The dotted blue lines indicate the 95% confidence intervals

**Fig. 2:**
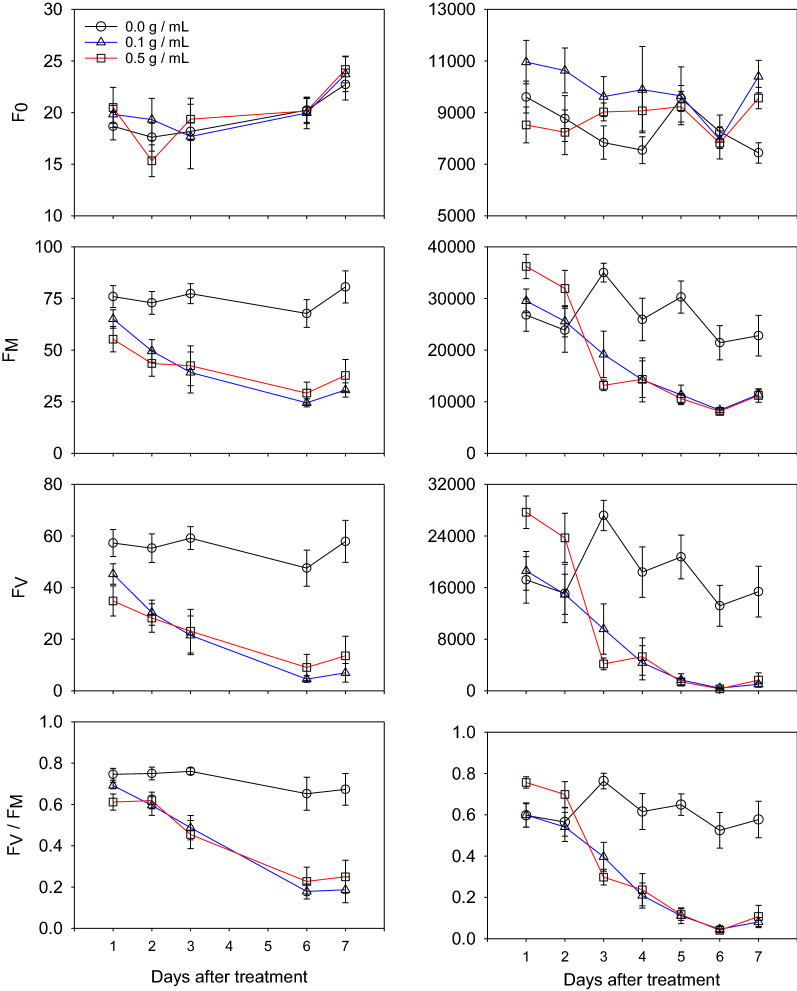
Line graphs of minimum fluorescence (F_0_), maximum fluorescence (F_M_), variable fluorescence (F_V_) and maximum photosynthetic efficiency (F_V_/F_M_) measured from imaging (**a**) and by handheld fluorometer (**b**) from 0.0, 0.1 and 0.5 g/mL applications of DCMU over 7 days. Symbols represent the average for each Day X Treatment (N = 4) whiskers represent standard error

The fluorescence values obtained from gantry imaging were highly correlated with those from the handheld fluorometer except for minimum fluorescence (F_0_) (Fig. 1)_._ The highest correlation observed between the handheld and imaging fluorescence was with the maximum photochemical efficiency (F_V_/F_M_) (*r* = 0.92) while the lowest observed correlation was for F_0_ (*r* = 0.02). Both variable fluorescence (F_V_) and maximum fluorescence (F_M_) also had high correlations, *r* = 0.70 and *r* = 0.64, respectively. The herbicide treatments produced wide ranges in fluorescence; for F_V_/F_M_ both the imaging and handheld data ranged from 0.1 to 0.8 and 0.05 to 0.8, respectively (Fig. 2). The treatments did not produce wide ranges of F_0_ with either the imaging or handheld data that ranged from 15 to 25 and 7000 to 12,000, respectively (Fig. 2). The observed ranges for F_V_/F_M_ and F_0_ represented approximately 8 and 1.5 times the minimum value, respectively.

### Comparison of time course fluorescence changes

**Fig. 3:**
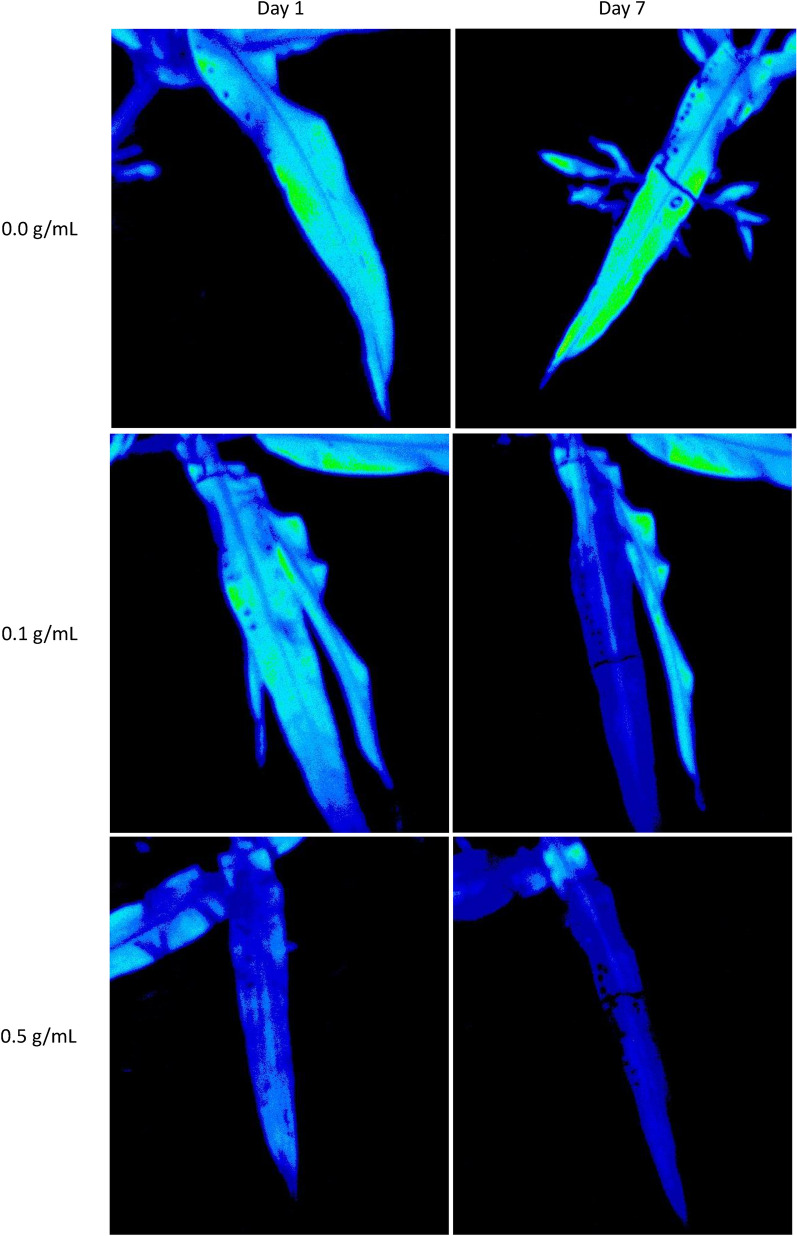
False-color images of sorghum leaves on days one and seven after being treated with DCMU at 2 different concentrations, 0.1 and 0.5 g/mL. Dark lines demarcate the leaves which received the treatments for visual referencing. Green represents more fluorescence whereas blue indicates tissue with lower fluorescence

The different treatment levels of DCMU did not cause measurable changes to fluorescence with either imaging or handheld instruments. However, the effects of the herbicide observed by both instruments and trends over the course of the experiment were similar (Fig. 2 and Table 1). The 0.5 g/mL treatment had higher F_V_/F_M_ compared to the 0.1 and 0.0 g/mL treatments on the 1st day for both instruments and both 0.5 and 0.1 g/mL treatments were lower than the 0.0 g/mL treatment on the 7th day for both instruments. Both methods agreed with respect to when observable differences between the control and herbicide treatments could be quantified. Both methods showed differences between the F_V_/F_M_ of the herbicide treatments relative to the control on the 2nd day through the 7th day. The imaging method had differences between F_M_ for the herbicide and control treatment on the 2nd day while the handheld had differences on the 3rd day (Fig. 2). The difference between treated and untreated leaves was clear when visible in the images (Fig. 3). The data from the imaging system also had less variability, as measured with standard error, compared to the handheld instrument. The imaging system, with the 4 m distance between measurements, was able to collect 24 measurements in under 18 min whereas the handheld fluorometer took under 10 min.

**Table 1:**
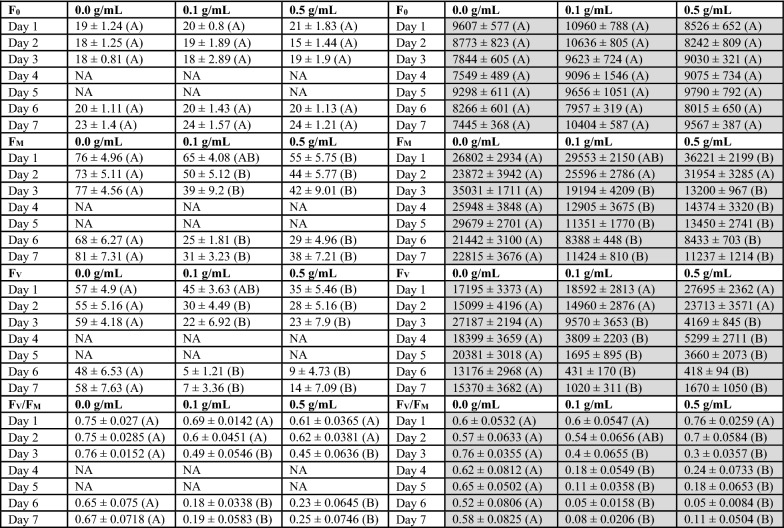
Mean ± standard error with statistical groupings for minimum fluorescence (F_0_), maximum fluorescence (F_M_), variable fluorescence (F_V_) and maximum photosynthetic efficiency (F_V_/F_M_) from Fig. 2

Means and standard errors calculated for each Treatment X Day (N = 8). Letters indicate statistical grouping based on Tukey comparison of least square means. Different letters indicate a significant difference between treatment for each day. Data from the fluorescence imaging is on the left of the table (White) and handheld fluorescence is on the right (Grey)

### Chlorophyll concentration

**Fig. 4:**
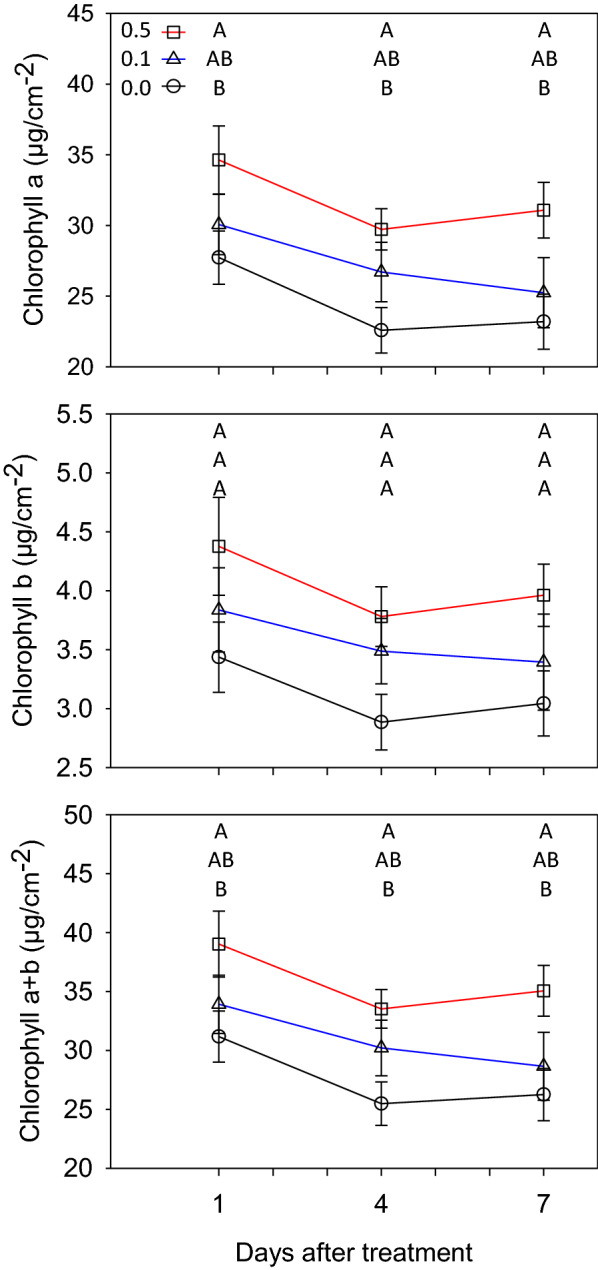
Line graphs of chlorophyll content from leaf samples taken on the 1st, 4, and 7th day after leaves were treated with the herbicide DCMU. Symbols indicate the mean for each Treatment × Day (N = 8) and the whiskers indicate standard error. Letters in each graph indicate Tukey assignments for each treatment within a day after treatment. Same letters indicate no significant difference

Herbicide treatment had a significant impact on chlorophyll concentration (Fig. 4 and Table 2). Chlorophyll concentration was highest for the 0.5 g/mL treatment on the 1st day with 34.6 µg/cm^−2^ and lowest for the control treatment at 27.7 µg/cm^−2^ and the 0.1 g/mL treatment at 30.7 µg/cm^−2^ (Fig. 3). On the 4th day, chlorophyll *a* concentration was highest for the 0.5 g/mL treatment at 29.7 µg/cm^−2^, lowest for the control treatment at 22.6 µg/cm^−2^ and the 0.1 g/mL treatment was between the other two treatment levels at 26.7 µg/cm^−2^. On the 7th day, chlorophyll concentration was highest for the 0.5 g/mL treatment at 31.1 µg/cm^−2^ and lowest with the 0.1 g/mL and control treatments of 25.2 and 23.2 µg/cm^−2^, respectively. Chlorophyll *b* concentrations followed a similar trend as chlorophyll *a* with the 0.5 g/mL treatment always being the highest on all days and the control always being the lowest.

**Table 2:** Analysis of variance (ANOVA) results from proc MIXED analysis with SAS for genotype treatment, days after treatment (DAT) and the two-way interactions between them for chlorophyll a (Chl a), chlorophyll b (Chl b) and total chlorophyll (Chl a + b)

Chl a			
Effect	DF	F value	Pr > F
Genotype	1	13.36	0.0005
Treatment	2	18.81	< .0001
DAT	3	7.15	0.0003
Genotype×Treatment	2	6.12	0.0034
Genotype×DAT	3	0.27	0.8477
Treatment×DAT	6	0.24	0.9613

Chl b			
Effect	DF	F value	Pr > F
Genotype	1	18.01	< .0001
Treatment	2	10.74	< .0001
DAT	3	1.93	0.1327
Genotype×Treatment	2	2.80	0.0673
Genotype×DAT	3	0.11	0.9565
Treatment×DAT	6	0.09	0.9972

Chl a + b			
Effect	DF	F value	Pr > F
Genotype	1	14.42	0.0003
Treatment	2	18.08	< .0001
DAT	3	6.26	0.0008
Genotype×Treatment	2	5.75	0.0047
Genotype×DAT	3	0.25	0.8621
Treatment×DAT	6	0.22	0.9688

## Discussion

Chlorophyll fluorescence is an important trait and useful method to study photosynthetic efficiency, which provides insight into the physiological status of the plant. Despite its ability to rapidly and nondestructively capture the status of the light-dependent reactions, the implementation for screening large plant populations in a field environment remains onerous. To overcome this burden, the deployment of chlorophyll fluorescence imaging systems in the field environment is greatly needed so that these data can be captured and utilized for both research and cultivar development. However, ensuring that accurate chlorophyll fluorescence data is being obtained by the imaging system is critical for future applications. The goal of the present study was to validate a PS2 fluorescence imaging system deployed on the TERRA-REF field scanalyzer with a commercial handheld fluorometer to assess the performance of the imaging system. Additionally, the imaging system was evaluated for its ability to track changes in fluorescence over time so that temporal dynamics of stress symptomology could be quantified.

### Chlorophyll fluorescence imaging correlation with a handheld fluorometer

The fluorescence imaging system was able to measure fluorescence and could quantify the effects of DCMU on photosynthesis. Previous studies have shown that DCMU, which blocks electron transport, directly impacts the light-dependent reactions of photosynthesis [24, 26]. Because of this, DCMU has been used to validate previous fluorescence imaging systems and why it was used in the present study [26–28]. With this in mind, sorghum leaves were treated with DCMU to evaluate a fluorescence imaging system deployed in a field environment. The high correlations observed between the handheld fluorometer and the fluorescence imaging system data for F_V_/F_M_, F_M_ and F_V_ provide evidence that the imaging system represents the state of the light-dependent reactions accurately. As expected, there was an extremely low correlation with minimum fluorescence (F_0_) because the herbicide treatment had no effect on F_0,_ and the ranges produced for F_0_ were extremely narrow compared to maximum fluorescence (F_M_). The high correlations observed with F_M_ and F_V_, with the inherent correlation of F_V_ being derived from F_0_ and F_M_, agreed with other studies on the effects of DMCU [39].

When the imaging data was treated like handheld data, i.e., only a small select portion of the image was used, the imaging data had a lower standard error. This level of data quality is especially important for use with field studies. Fluorescence data obtained from the full canopy of a plot, given the present results, would likely be less variable compared to obtaining multiple point measurements from a handheld device over the canopy. Additionally, capturing the canopy fluorescence of field plots with a handheld device would be time-consuming and nearly impossible from an entire field with hundreds of experimental plots, whereas the imaging system can obtain image data from all the plots in the field in hours. However, the use of fluorescence imaging in a field setting is challenging and as with the present study, conditions were not always optimal. A low wind speed is necessary to provide stability for the imaging system itself and for the plants being imaged. Additional experiments will be needed with the system to dissect the spatial variation of a leaf’s fluorescence with handheld devices to compare with image data.

### Application of imaging system to timescale experiments

The similarity between the fluorescence response over the days that fluorescence images were able to be collected, and the handheld data, indicate that the system can correctly track fluorescence changes in plants over time. Few datasets exist from field-grown plants with field-deployed imaging and make it difficult to compare the present results. Fluorescence imaging has been used previously to track the effects of fungal pathogens on photosynthesis. The fluorescence imaging showed the effects of the fungal infection on the leaf prior to visible phenotypes [40]. Fluorescence imaging was able to track the reduction of photosynthesis over 7 days as the fungal infection continued to reduce photosynthesis at inoculation sites. As the infection began to spread through the leaf, fluorescence imaging was also able to visualize the reduction of photosynthesis in areas surrounding the inoculation site [40]. Additional studies using fluorescence imaging have tracked changes over time in response to drought [41, 42] and chilling [43]. The present study was also able to track photosynthetic reduction over 7 days and with comparable results to a handheld fluorometer. Given this, the gantry-based imaging system, under appropriate environmental conditions, can track changes in fluorescence over daily and weekly timescales. Because of the adverse effects that wind can have on the imaging process, if an experiment requires a strict timeline of measurements additional steps would need to be taken to ensure that imaging conditions would be optimal on days requiring data collection.

### Chlorophyll and chlorophyll fluorescence response

The accumulation of chlorophyll with the highest herbicide rate was unexpected, however the observed accumulation of chlorophyll was not associated with an increase in maximum chlorophyll fluorescence. It is possible, given the nutrient status of the plants from the fertilizer applications, that the plants attempted to compensate for reduced photochemical efficiency by increasing the amount of chlorophyll available for capturing light energy. Previous studies have shown high concentrations of DCMU had either no effect on chlorophyll synthesis or a slight decrease [44, 45]. However, these studies used algae and were performed in a lab setting, conditions in stark contrast to the present experiment. Further studies are needed to determine DCMU effects on chlorophyll synthesis in sorghum with regards to concentration. Despite an increase of chlorophyll, the imaging system was able to quantify the reduction of photochemical efficiency.

## Conclusion

The results from the present study have shown the ability of the imaging system from the TERRA-REF gantry phenotyping system to provide precise and accurate chlorophyll fluorescence data. The high correlation observed between the handheld fluorometer and imaging system for F_V_/F_M_ provides evidence that the system can provide accurate chlorophyll fluorescence measurements from plants in the field. Utilization of the system to identify genetic information about chlorophyll fluorescence will be undertaken in future field experiments. These studies will also be leveraged with additional sensor data and information to identify how chlorophyll fluorescence relates to physiological processes.

## Methods

### Planting and growth conditions

**Fig. 5:**
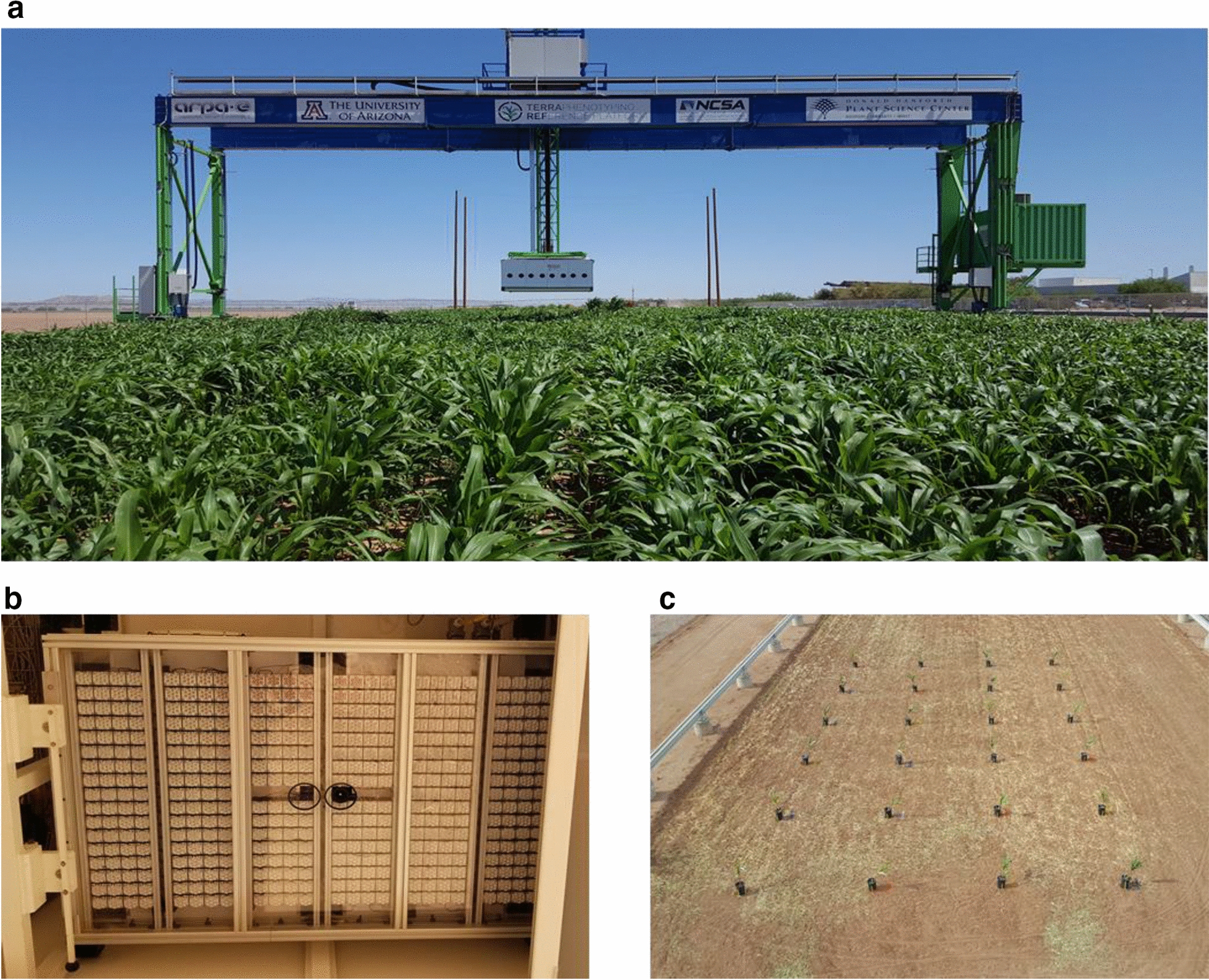
**a** The TERRA-REF field scanalyzer located at the Maricopa Agricultural Center. The white box suspended from the cross member houses the sensor payload including the PS2 imaging system. **b** The PS2 imaging system consists of a bank of light-emitting diodes (LEDs) to provide the saturating light flash. The camera is located in the center of the LED array (small black circle). **c** Aerial image of the 24 potted sorghum plants within the field where the scanalyzer resides courtesy of Maria Newcomb. The pots were spaced 4 m from each other in each direction and organized in a completely randomized design. Plant residue from the previous crop remained in the field

Two commercial grain sorghum [*Sorghum bicolor* (L.) Moench] hybrids, P-86Y89 and P-848G62 (DuPont Pioneer, Johnston, IA, USA) were evaluated at the Maricopa Agricultural Center (MAC) of the University of Arizona located in Maricopa, AZ (33°04′37″ N, 111°58′26″ W, elevation 358 m) in the spring of 2019. For each genotype, three seeds were sown into 12, 18.9 L pots filled with Sunshine Mix (Sun Gro Horticulture Agawam, MA, USA) potting soil at a depth of approximately 2.54 cm on February 17, 2019, giving a total of 24 pots for the experiment. At 14 days after planting (DAP), plants were thinned for uniform growth and height to one plant per pot. Plants were maintained in a greenhouse where they were watered every 4 to 5 days while vegetative growth continued. At 35 DAP, plants were transported outside to acclimate to field conditions. After 16 days of acclimation (51 DAP), plants were moved to the field with the field-scanalyzer phenotyping system so that imaging data could be collected (Fig. 5). The potted plants were arranged in a completely randomized design with four replications each of the three treatment levels explained below. Each potted plant was spaced four meters away from the nearest neighbor to ensure that no light emitted from the PS2 imagining system affected the dark-adapted state of the plants when chlorophyll fluorescence measurements were taken at night. While plants were in the field, they were watered every 2–3 days as needed to ensure plants were not water stressed. Every 2 weeks, plants received a water-soluble fertilizer (Nutriculture 20-20-20, Plant Marvel Laboratories Inc., Chicago Heights, IL, USA) with normal watering.

### Herbicide treatment

The herbicide 3-(3′,4′-dichlorophenyl)-1,1-dimethylurea (DCMU) was used to inhibit photosynthesis in the leaves of the sorghum plants to provide attenuated levels of chlorophyll fluorescence for imaging purposes. To achieve contrasting levels of chlorophyll fluorescence, a pilot trial was conducted (data not published) with varying rates of DCMU (Diuron 80DF Alligare LLC., Opelika, Alabama, USA) applied to sorghum leaves. The treated leaves were then measured for several days after the DCMU application using the handheld fluorescence instrument described below. Rates of 0.1 and 0.5 g/mL were found to give the best separation in chlorophyll fluorescence values and were used for the present study. For each plant, the uppermost fully expanded leaf was marked using a paint pen approximately 20 cm from the mainstem. One mL of each treatment level [0.0 (control level), 0.1, 0.5 g/mL] was applied directly to the leaf surface distal to the paint pen mark and then spread toward the tip of the leaf using a paper towel. Treatments were applied 51 DAP between the hours 14:00 and 16:00 (MST). The following morning at approximately 8:00 (MST) all the marked leaves were wiped with deionized water to remove any residual surfactant.

### Chlorophyll fluorescence

Handheld measurements of chlorophyll fluorescence (CF) were obtained with a Fluorpen Z995-PAR (Qubit systems INC, Kinston Ontario, Canada). For measurement collection, a leaf clip was attached to each of the treated leaves approximately 10 cm above the paint pen mark to the side of the leaf midrib. Data was obtained using the fluorescence transient (OJIP) protocol, which provides a saturating flash and allows calculation of F_V_/F_M_. Data were collected at 23:30 (MST) for 7 days after treatment application.

The chlorophyll fluorescence imaging system used in this study is one of the integrated sensors carried by the LemnaTec Scanalyzer which is part of the TERRA-REF phenotyping project (www. terraref.org). The imaging system is comprised of an LED panel that provides a saturating flash of 7000 µmol photosynthetically active radiation (PAR) to the imaging area and a Manta G-235b (Allied Vision Technologies GmbH, Stadtroda, Germany) camera. For each measurement event, the system executes the following protocol for collecting data: one image is taken prior to the saturating flash of LED light; the saturating flash of LED light is emitted; 50 images are then taken during the one-second pulse of light; and 50 images are taken after the pulse of light. The imaging system was positioned 70 cm above the target leaves. Chlorophyll fluorescence imaging data was collected on the 1st, 2nd, 3rd, 6 and 7th night after herbicide treatments were applied from 21:30 till 22:30 (MST). On the 4 and 5th days the wind speed was too high (> 5 mph) to collect data. Sunset occurred at 19:00 which provided the plants a minimum of 2 and a half hours to dark adapt. Acceptable dark adaptation is between 15–20 min [46].

For image analysis, binary (BIN) files from the system were converted to portable gray map (PGM) files with ImageJ [47]. A small circle, approximately 3 mm in diameter, a similar size as the clip used for handheld measurements, was used to extract fluorescence intensity from each grey scale image in the approximate area that handheld measurements were obtained. If this area was not visible in the image, the nearest area was used. The second image, the first image after start of flash, was used as the minimum fluorescence (F_0_) value and the highest fluorescence value from the subsequent images was used as the maximum fluorescence (F_M_). Variable fluorescence was calculated by subtracting F_0_ from F_M_. Maximum photosynthetic efficiency (F_V_/F_M_) was calculated by dividing F_V_ by F_M_.

### Chlorophyll quantification

To ensure that the imaging system was capable of measuring changes in the efficiencies of the light reactions and not just the amount of chlorophyll present, chlorophyll concentrations were quantified from leaves on the 1st, 4, and 7th days after application of the herbicide. Two sets of 3 leaf discs (0.3 cm^−2^) were collected from each plant at midday into 1.2 mL polypropylene tubes and kept on ice in the field until they could be stored at − 80 °C in the laboratory. Chlorophyll was extracted by adding 1 mL of cold (4 °C) 100% methanol (Fisher Scientific, Waltham, MA) to the polypropylene tubes. Samples were covered and placed in an orbital shaker (Stovall Life Sciences, Greensboro, NC) on the max setting (~ 1.5 rotations per second) and kept at 4 °C for 48 h. Each morning (~ 8:00 MST) and each afternoon (~ 16:00 MST) the samples were inverted 5 times then placed back in the orbital shaker. After 48 h, 200 µL of the methanol now containing the chlorophyll, from each sample was transferred to a clear, 96-well flat bottom microplate (Fisher Scientific, Waltham, MA, USA). Sample fluorescence was measured using a Synergy HT (BioTek Instruments, Winooski, VT, USA) plate reader with 665 and 652 nm wavelengths. A correction factor for each wavelength was developed by BioTek for the 96-well microplate. Chlorophyll concentration, in µg/mL, from each sample was calculated following Porra et al. [48] where Chl *a* (μg/mL) = 16.29 A_665.2_–8.54 A_652_, Chl *b* (μg/mL) = 30.66 A_652_–13.58 A_665.2_ and, Chl *a* + *b* (μg/mL) = 22.12 A_652_ + 2.71 A_665.2_. The µg/mL was divided by the total leaf area per sample (0.848 cm^−2^) to get µg/cm^−2^.

### Statistics

To compare the chlorophyll fluorescence phenotypes from the image-based method and a handheld device values for F_0_, F_M_, F_V_, F_V_/F_M_ from each Genotype × Treatment × Day were used to assess the degree of association between the handheld and imaging systems. The Pearson’s correlation coefficients (*r*) and associated *p*values (*p*) were calculated using the CORR procedure in SAS. The results were graphed with SigmaPlot v14.0 (Systat Software Inc., San Jose, CA, USA).

To determine if the imaging system was capable of capturing the temporal effect of the treatments on chlorophyll fluorescence a repeated measures mixed linear model was fitted to the data for each trait (F_0_, F_M_, F_V_, F_V_/F_M_) for both instruments using SAS v9.4 (SAS Institute Inc., Cary, NC). The model was as follows:

$${\text{Y}}_{ijk} = \, \mu \, + {\text{ genotype}}_{i} + {\text{ treatment}}_{j} + {\text{ day}}_{k} + \, \left( {{\text{teatment }} \times {\text{ day}}} \right)_{jk} + \, \left( {{\text{genotype }} \times {\text{ treatment}}} \right)_{ij} + \, \left( {{\text{genotype }} \times {\text{ day}}} \right)_{{{\text{ik}}}} + \varepsilon_{ijk}$$

with $\varepsilon_{ijkl}$ equal to $Var(\varepsilon_{ijkl} ) = \sigma^{2} Cov(\varepsilon_{ijkl} ,\varepsilon_{i^\prime jkl} ) \, = \, \rho \, \sigma^{2} , \, i \, \ne \, i^\prime$.

in which Y_*ijk*_ is the single plant-level measurement; µ is the grand mean; genotype_*i*_ is the effect of the *i*th genotype; treatment_*j*_ is the effect of the *j*th DCMU herbicide treatment; day_*k*_ is the effect of the *k*th day on which measurements were taken; (treatment × day)_*jk*_ is the interaction effect between the *j*th treatment level and the *k*th day; (genotype × treatment)_*ij*_ is the interaction effect of the *i*th genotype and the *j*th treatment level; (genotype × day)_ik_ is the interaction effect of the *i*th genotype and *k*th day; and ε_*ijkl*_ is the random error term following a normal distribution with mean 0 and variance *σ*^*2*^. The residual variance, ε_*ijkl*_, was modeled using a correlated error variance structure that incorporated a constant, non-zero, correlation term (*ρ*) among error terms to account for correlation among the days on which measurements were taken on the same experimental unit—the individual plants. All terms were fitted as fixed effects. Tests of fixed effects were conducted using the Kenward Roger approximation for the calculation of degrees of freedom.

## Data Availability

The data can be made available upon reasonable request and by mailing us an external drive.

## Abbreviations

DCMU: 3-(3′,4′-dichlorophenyl)-1,1-dimethylurea
F_M_: Maximum fluorescence
F_0_: Minimum fluorescence
F_V_: Variable fluorescence
F_V_/F_M_: Maximum photochemical efficiency
PS2: Photosystem II
DAP: Days after planting
LED: Light emitting diode
FOV: Field of view
DAT: Days after treatment
